# Increased risk of brain cancer incidence in stroke patients: a clinical case series, population-based and longitudinal follow-up study

**DOI:** 10.18632/oncotarget.22480

**Published:** 2017-11-15

**Authors:** Chih-Wei Chen, Tain-Junn Cheng, Chung-Han Ho, Jhi-Joung Wang, Shih-Feng Weng, Ya-Chin Hou, Hung-Chi Cheng, Chung-Ching Chio, Yan-Shen Shan, Wen-Tsan Chang

**Affiliations:** ^1^ Institute of Clinical Medicine, College of Medicine, National Cheng Kung University, Tainan 701, Taiwan; ^2^ Division of Neurosurgery, Department of Surgery, Chi Mei Foundation Medical Center, Tainan 710, Taiwan; ^3^ Department of Occupational Safety and Health/Institute of Industrial Safety and Disaster Prevention, College of Sustainable Environment, Chia Nan University of Pharmacy and Science, Tainan 717, Taiwan; ^4^ Department of Neurology and Occupational Medicine, Chi Mei Foundation Medical Center, Tainan 710, Taiwan; ^5^ Department of Medical Research, Chi Mei Foundation Medical Center, Tainan 710, Taiwan; ^6^ Department of Hospital and Health Care Administration, Chia Nan University of Pharmacy and Science, Tainan 717, Taiwan; ^7^ Department of Anesthesiology, Chi Mei Foundation Medical Center, Tainan 710, Taiwan; ^8^ Department of Health Care Administration and Medical Informatics, Kaohsiung Medical University, Kaohsiung 807, Taiwan; ^9^ Department of Biochemistry and Molecular Biology, College of Medicine, National Cheng Kung University, Tainan 701, Taiwan; ^10^ Division of General Surgery, Department of Surgery, College of Medicine, National Cheng Kung University, Tainan 701, Taiwan

**Keywords:** stroke, ischemic stroke, brain cancer, glioblastoma mutiforme (GBM), nationwide population-based cohort

## Abstract

Stroke and brain cancer are two distinct diseases. However, the relationship between both diseases has rarely been examined. This study investigated the longitudinal risk for developing brain cancer in stroke patients. To study this, we first reviewed the malignant gliomas previously with or without stroke using brain magnetic resonance imaging (MRI) images and the past histories. Two ischemic stroke patients before the malignant glioma were identified and belonged to the glioblastoma mutiforme (GBM). Particularly, both GBM specimens displayed strong hypoxia-inducible factor 1α (HIF-1α) expression in immunohistochemical (IHC) staining. To elucidate the significance of this relationship, we then used a nationwide population-based cohort in Taiwan to investigate the risk for the incidence of brain cancer in patients previously with or without stroke. The incidence of all tumors in the stroke group was lower than that in the control group with an adjusted hazard ratio (HR) of 0.79 (95% confidence interval [CI]: 0.74-0.84) in both gender and age older than 60 years. But the stroke patients had higher risk of developing only brain cancer with an adjusted HR of 3.09 (95% CI: 1.80-5.30), and otherwise had lower risk of developing head and neck, digestive, respiratory, bone and skin, as well as other tumors, all with p<0.05. After stratification by gender and age, the female and aged 40-60 year old stroke patients had higher risk of developing brain cancer with an adjusted HR of 7.41 (95% CI: 3.30-16.64) and 16.34 (95% CI: 4.45-62.13), respectively, both with p<0.05. Patients with stroke, in particular female and age 40-60 years old, have an increased risk for developing brain cancer.

## INTRODUCTION

Primary brain tumors are the leading causes of cancer-related deaths worldwide [1]. The annual global and age-standardized incidence of primary malignant brain tumors is 3.7 per 100,000 for males and 2.6 per 100,000 for females [2, 3]. In Taiwan, about 1000 patients are diagnosed annually with primary brain cancers, 60% to 70% of which are malignant gliomas [4]. The malignant brain tumors account for 1% of all newly diagnosed adult cancers and 2% of cancer-related deaths [5]. The only established risk factors for brain tumors are high-dose ionizing radiation and certain rare genetic alterations, which together explain only a small proportion of cases [6–8]. Therefore, it is worthwhile to search for the possible risk factors to decrease disease occurrence.

Stroke is the most common disease of complex disability and the third leading cause of death in Taiwan. Its annual rate of age-standardized mortality is steadily declining between 2001 and 2012 [9]. The most common type of stroke is ischemic stroke, with the majority of subtypes related to small vessel occlusion. It has been suggested that hypertension, diabetes, hyperlipidemia, obesity, and smoking are important risk factors to stroke morbidity [10]. Chen *et al.* previously analyzed the National Health Insurance Research Database (NHIRD) of Taiwan to examine the prevalence and mortality of stroke [11]. The data revealed that the stroke mortality increased in aged individuals with a higher prevalence among females than males.

Stroke and brain cancer are two distinct and unique diseases. The risk for ischemic stroke is higher in patients with gliomas, reaching up to 9%, compared to the general population (2.7%) [12–14]. The diagnosis of an ischemic cerebrovascular accident (CVA) is rare in the initial presentation of glioma [12, 15]. The onset of neurological deficits in patients with glioma could be secondary to tumor progression, brain edema, seizures and stroke [16]. Stroke has been described more frequently as a postoperative complication and a late complication of radiotherapy, and also associated with tumor-induced hypercoagulability or nonbacterial thrombotic endocarditis [13, 17]. However, there are a handful of studies describing stroke as a risk factor to induce brain tumor. Schlehofer *et al.* found that stroke can increase the risk of glioma [18]. They showed a combined odds ratio (OR) of 1.9 for self-reported stroke occurring less than 2 years before diagnosis of either meningioma or glioma.

It has been shown that cancer patients are at an increased risk of stroke incidence [19–21]. In addition, recent studies suggested that the ischemic stroke survivors have an increased incidence of developing tumor [22]. Thus, we hypothesized that the stroke is in relation with the malignant glioma development. To examine this possibility, we first retrospectively reviewed the malignant glioma cases previously with or without stroke using brain MRI images and the past histories diagnosed in the Chi Mei Foundation Medical Center. Furthermore, to explore the potential mechanism for developing malignant glioma in stroke patients, we investigated the expression of hypoxia key regulator HIF-1α using IHC staining in the tumor specimens surgically removed from the malignant glioma patients previously with or without stroke. Moreover, to elucidate the significance of this relationship, we then used a nationwide population-based cohort in Taiwan to investigate the risk of brain cancer incidence in patients previously with or without stroke. In the meantime, we also analyzed the new incidence of other tumors in patients previously with or without stroke.

## RESULTS

### Ischemic stroke increases the incidence of malignant glioma

**Figure 1:**
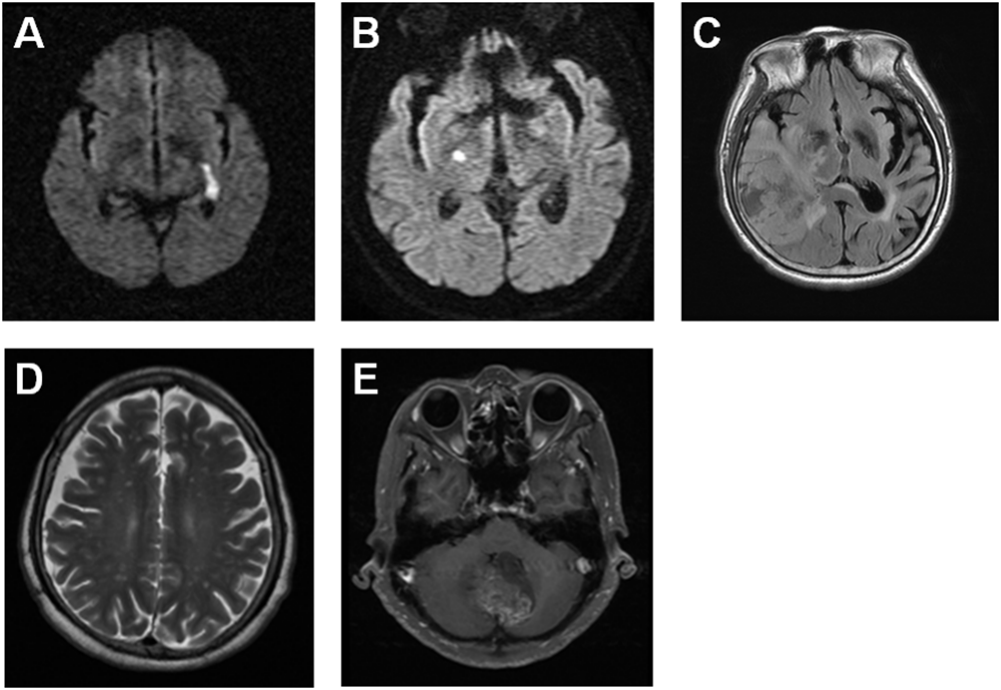
Two GBM cases are diagnosed previously with ischemic stroke using brain MRI images The brain MRI images indicate a 72 year-old male patient with the left internal capsule acute infarction on January 21, 2007 **(A)** and the right hypothalamus acute infarction on April 2, 2009 **(B)**, as well as the right temporal GBM on June 21, 2016 **(C)**. The brain MRI images reveal a 67 year-old female patient with the bilateral frontoparietal subcortical old infarction **(D)** and the cerebellar vermis GBM on May 21, 2015 **(E)**.

We retrospectively reviewed malignant glioma cases using brain MRI images and the past histories diagnosed in the Chi Mei Foundation Medical Center. There were 21 GBMs (WHO grade IV), 8 anaplastic astrocytomas (WHO grade III), 1 grade II astrocytoma, and 3 grade I astrocytomas. Two patients (6%) with ischemic stroke before the malignant glioma were diagnosed and belonged to the group of 21 GBMs. One case is a 72 year-old male with first left internal capsule acute infarction on January 21, 2007 and second right hypothalamus acute infarction on April 2, 2009 (Figure 1A and 1B). The patient received clinical follow up but right temporal GBM was diagnosed on June 21, 2016 (Figure 1C). Another case is a 67 year-old female with history of ischemic stroke about 5 years ago and cerebellar vermis GBM was diagnosed on May 21, 2015 with the image revealing cerebellar GBM with bilateral frontoparietal subcortical old infarction (Figure 1D and 1E). These results reveal a higher possibility of stroke in relation with developing malignant glioma.

### Ischemic stroke increased the incidence of malignant glioma is accompanied with HIF-1α signaling

**Figure 2:**
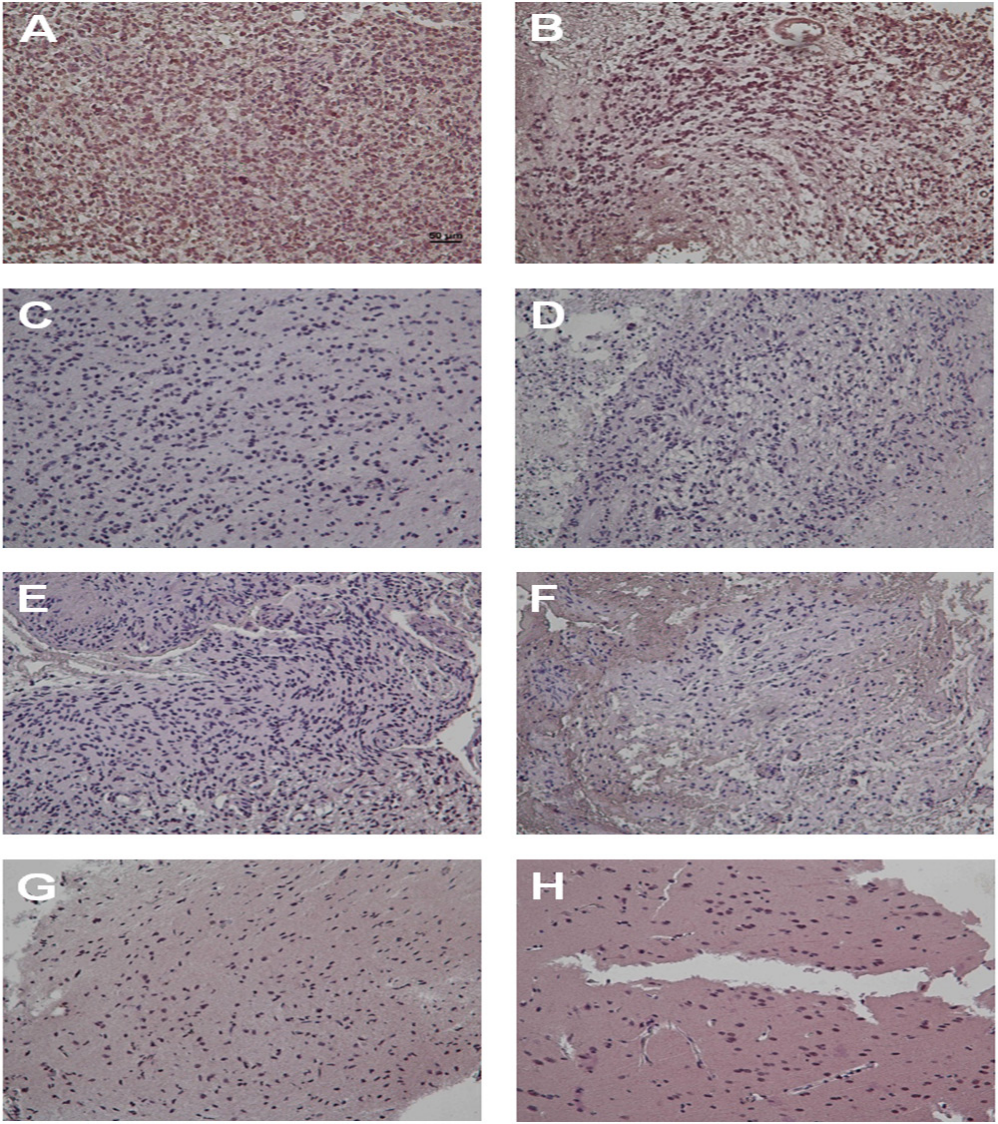
Tumor specimens from GBM patients previously with ischemic stroke display strong HIF-1α expression by IHC staining The IHC staining images indicate the expression level of HIF-1α in GBM patients previously with ischemic stroke **(A** and **B)**, GBM patients previously without stroke **(C, D** and **E)** and grade I astrocytoma patients **(F, G** and **H)**.

Tumor specimens removed from 8 patients (2 GBMs previously with stroke, 3 GBMs previously without stroke and 3 grade I astrocytomas) with histologically-proven, surgically-treated malignant glioma were examined by IHC staining for HIF-1α, a key hypoxic regulator. Strong staining was observed in 100% (2/2) of GBMs previously with stroke (Figure 2A and 2B). In contrast, no staining was observed in 100% (3/3) of GBMs previously without stroke and 100% (3/3) of grade I astrocytomas (Figure 2C–2H). Compared with the malignant glioma previously without stroke, the HIF-1α expression significantly increased in the GBM previously with stroke. These results indicate that the HIF-1α play an important role in ischemic stroke inducing the development of GBM.

### Increased the incidence of brain cancer is associated with stroke

To investigate the significance of this relationship, we then used a nationwide population-based cohort in Taiwan to analyze the risk for developing brain cancer in patients previously with or without stroke. A total of 17161 patients in the stroke group and 68644 patients in the control group were enrolled. Both groups consisted of 58.50% men and 41.50% women. The baseline characteristics and comorbidities of the stroke patients and control group were summarized in Table 1. The stroke group had higher prevalence of diabetes mellitus (DM) (31.73% *vs*. 9.36%), hypertension (HTN) (67.63% *vs*. 22.55%), hyperlipidemia (18.03% *vs*. 5.79%), coronary artery disease (CAD) (21.40% *vs*. 7.73%), and chronic kidney disease (CKD) (8.19% *vs*. 2.12%), all with *p* < 0.0001 (Table 1).

**Table 1:** Demographic characteristics and comorbid medical disorders for stroke and control groups

Characteristics	Stroke	Control	P-value^*^
	N=17161	N=68644	
Age, N (%)			
≦40	688 (4.01)	2752 (4.01)	1.0000
40-60	4116 (23.98)	16464 (23.98)	
60-80	10002 (58.28)	40008 (58.28)	
>80	2355 (13.72)	9420 (13.72)	
Gender, N (%)			
Male	10039 (58.50)	40156 (58.50)	1.0000
Female	7122 (41.50)	28488 (41.50)	
Comorbidity, N (%)			
Diabetes Mellitus	5445 (31.73)	6422 (9.36)	<0.0001
Hypertension	11606 (67.63)	15476 (22.55)	<0.0001
Hyperlipidemia	3094 (18.03)	3972 (5.79)	<0.0001
Coronary artery disease	3672 (21.40)	5306 (7.73)	<0.0001
Chronic kidney disease	1405 (8.19)	1453 (2.12)	<0.0001

^*^P-value was derived from Pearson's chi-square.

**Figure 3:**
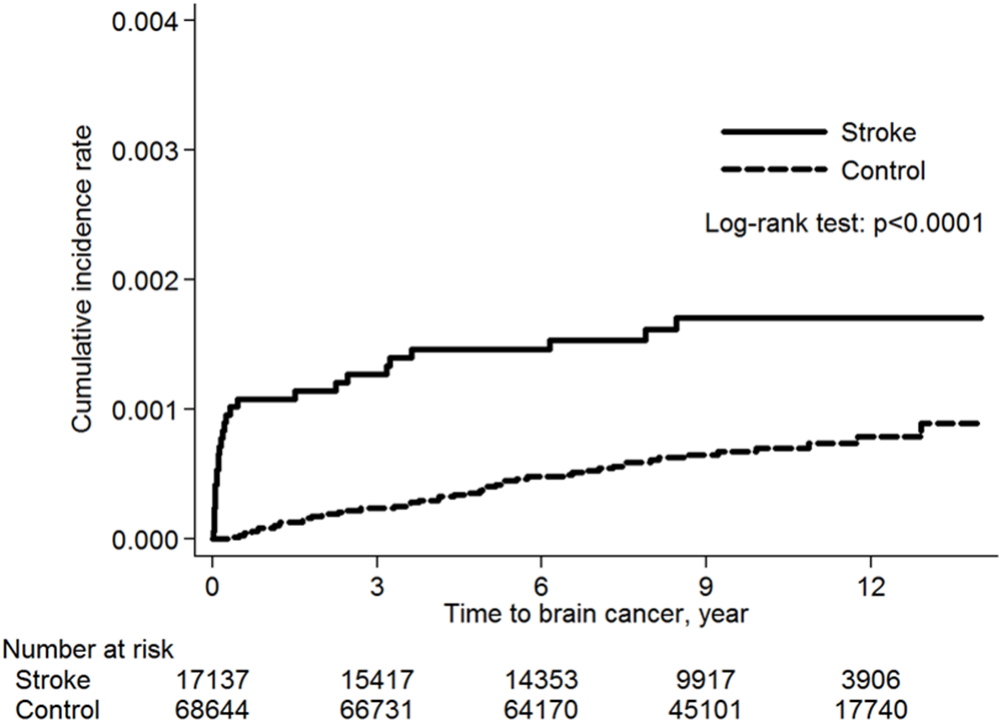
The Kaplan-Meier analysis of brain cancer free in stroke and control groups

The incidence of all cancers was 9.43 per 1000 person-year in the stroke group, slightly lower than that (10.16 per 1000 person-year) in the control group with a crude hazard ratio (HR) of 0.93 (95% confidence interval [CI]: 0.88-0.98) (Table 2). After adjusting for risk factors, we found that the stroke patients had lower risk for developing any tumor, with an adjusted HR of 0.79 (95% CI: 0.74-0.84) in both gender and age older than 60 years (Table 2). However, the stroke patients had higher risk of developing only brain cancer with an adjusted HR of 3.09 (95% CI: 1.80-5.30). Otherwise, this group had lower risk of developing head and neck cancer with adjusted HR of 0.89 (95% CI: 0.67-1.18), digestive cancer with adjusted HR of 0.75 (95% CI: 0.70-0.82), respiratory cancer with adjusted HR of 0.85 (95% CI: 0.73-0.99), bone and skin cancer with adjusted HR of 0.69 (95% CI: 0.48-0.98) and other cancers with adjusted HR of 0.74 (95% CI: 0.63-0.89), all with *p* < 0.05 (Table 3). The Kaplan-Meier survival analysis showed a difference in the incidence rates of brain cancer between the stroke and control groups (*p*<0.0001 for the log-rank test) over time (Figure 3).

**Table 2:** The risks of developing cancers in the stroke and control groups

Characteristics	Stroke				Control				Crude HR^$^ (95% CI)	Adjusted HR^b^ (95% CI)
	N	Cancer	#PY	Rate^a^	N	Cancer	#PY	Rate^a^		
All	17161	1408	149263.46	9.43	68644	6763	665975.77	10.16	0.93 (0.88-0.98)^*^	0.79 (0.74-0.84)^*^
Age										
≦40	688	22	6475.73	3.40	2752	46	28976.48	1.59	2.15 (1.29-3.57)^*^	1.79 (0.99-3.23)
40-60	4116	276	38214.12	7.22	16464	1116	166239.69	6.71	1.08 (0.95-1.23)	0.90 (0.77-1.05)
60-80	10002	975	86312.45	11.30	40008	5015	382530.71	13.11	0.86 (0.80-0.92)^*^	0.76 (0.71-0.82)^*^
>80	2355	135	18261.16	7.39	9420	586	88228.89	6.64	1.08 (090-1.31)	0.73 (0.60-0.90)^*^
Gender										
Male	10039	946	85478.29	11.07	40156	4464	385779.52	11.57	0.96 (0.89-1.02)	0.81 (0.75-0.88)
Female	7122	462	63785.17	7.24	28488	2299	280196.25	8.21	0.88 (0.80-0.98)^*^	0.74 (0.67-0.83)

#PY: person-years.

^$^HR: hazard ratio.

^a^Rate: per 1000 person-years.

^b^Adjusted by age groups, gender and comorbidities.

^*^P-value<0.05.

**Table 3:** Crude and adjusted hazard ratio (HR) for the development of cancer among stroke and control groups

Cancer type	Stroke N=17161		Control N=68644		Crude HR (95% CI)	Adjusted HR^a^ (95% CI)
	N	%	N	%		
All (140–192)	1408	8.20	6763	9.85	0.93 (0.88-0.98)^*^	0.79 (0.74-0.84)^*^
Head and neck (140–149)	76	0.44	334	0.49	1.00 (0.78-1.28)	0.89 (0.67-1.18)
Digestive (150–159, 179, 185-189)	833	4.85	3979	5.80	0.93 (0.86-1.00)	0.75 (0.70-0.82)^*^
Respiratory (160–165)	235	1.37	1167	1.70	0.89 (0.77-1.02)	0.85 (0.73-0.99)^*^
Bone and Skin (170-173, 176)	43	0.25	233	0.34	0.81 (0.59-1.13)	0.69 (0.48-0.98)^*^
Brain cancer (191, 192.0, 192.1)	27	0.16	47	0.07	2.48 (1.55-3.98)^*^	3.09 (1.80-5.30)^*^
Others	194	1.13	1003	1.46	0.85 (0.73-0.99)^*^	0.74 (0.63-0.89)^*^

^a^Adjusted by age groups, gender and comorbidities.

^*^P-value<0.05.

The median time to develop any cancer was 4.08 years with interquartile range (IQR) from 1.42 to 6.98 in the stroke group, which is statistically significantly shorter than that of 5.05 years (IQR: 2.37-7.78) in the control group with *P*<0.0001. The median time to develop brain cancer was 0.18 years (IQR: 0.05-2.46) in the stroke group, which is also statistically significantly shorter than that of 4.61 years (IQR: 1.80-7.04) in the control group with *P*<0.0001. After stratified by gender and age, two groups were notable for higher risk of developing brain cancer: female stroke patients with an adjusted HR of 7.41 (95% CI: 3.30-16.64), and age 40-60 year old stroke patients with an adjusted HR of 16.34 (95% CI: 4.45-62.13), both with *p* < 0.05 (Table 4).

**Table 4:** The risks of brain cancer in stroke and control groups stratified by age and gender

Characteristic	Stroke				Control				Crude HR (95% CI)	Adjusted HR^b^ (95% CI)
	N	Cancer	#PY	Rate^a^	N	Cancer	#PY	Rate^a^		
All	17161	27	155230.90	0.17	68644	47	683868.69	0.07	2.48 (1.55-3.98)^*^	3.09 ( 1.80-5.30)^*^
Age										
≦40	688	5	6538.55	0.76	2752	--	29133.61	--	--	--
40-60	4116	7	39321.49	0.18	16464	4	169615.42	0.02	7.40 (2.17-25.29)^*^	16.34 (4.45-62.13)^*^
60-80	10002	13	90470.92	0.14	40008	39	395503.55	0.10	1.44 (0.77-2.70)	1.67 (0.83-3.36)
>80	2355	2	18899.95	0.11	9420	4	89616.11	0.04	2.23 (0.41-12.16)	0.92 (0.16-5.31)
Gender										
Male	10039	13	89349.63	0.15	40156	32	397677.55	0.08	1.78 (0.93-3.38)	1.57 (0.76-3.25)
Female	7122	14	65881.28	0.21	28488	15	286191.14	0.05	3.97 (1.92-8.22)	7.41 (3.30-16.64)^*^

#PY, person-years.

^a^Rate: per 1000 person-years.

^b^Adjusted by age groups, gender and comorbidities.

^*^P-value<0.05.

## DISCUSSION

There is an increased interest in elucidating the epidemiologic causes of brain tumors. Many epidemiologic studies have focused on glioma but most of them have had limitations. For the relatively rare cases of brain cancers, most of the investigations are designed as case-control studies, which are susceptible to information bias. In addition, since glioma has a very poor prognosis, case-control studies are subjected to possible biases from the use of proxies for patients who are too sick to be interviewed, and from the effects of the illness on recall of past events. Moreover, some of the cohort studies have had only small numbers of cases [23, 24]. A large sample-sized cohort study is capable of overcoming the potential biases present in previous studies.

Primary brain tumors are the leading causes of cancer-related deaths worldwide [1]. In addition, stroke is a leading cause of functional dependence and death [25]. Brain cancer and stroke are two different and unique diseases, and there is no direct evidence of correlation between brain tumors and stroke. Nevertheless, it has been shown that cancer patients are at an increased risk of stroke incidence [19–21]. In addition, recent studies suggested that the ischemic stroke survivors have an increased incidence of developing tumor [22]. To the best of our knowledge, this report is the first nationwide population-based cohort study to investigate the risk of brain cancer among stroke patients in an Asian population. Our data revealed that stroke patients had higher risk for developing brain tumor with an adjusted HR of 3.09 (95% CI: 1.80-5.30) during the 14-year follow-up period, after adjusting for age and medical comorbidities (Table 3).

It is interesting that the stroke patients had higher risk of developing only brain cancer, but otherwise had lower incidence of developing other tumors such as head and neck, digestive, respiratory, or bone and skin cancers (Table 3). The possible explanation is that following a stroke, patients may modify aspects of their life styles such as diet control, regular exercise and sleep, so that the incidence of cancer after the stroke is diminished. In addition, there is probably a survival effect from the shorter duration of follow-up in stroke patients. In contrast, previous studies showed the annual rate of age-adjusted cancer incidence is higher among ischemic stroke patients compared with those in the general population [22]. This difference may result from the selection bias of stroke patients with less severity and the short follow-up years in developing cancer. In addition, the explanation underlying the increased risk of tumor in stroke survivors in all age groups is not fully known.

The median age at diagnosis for all primary brain and central nervous system (CNS) tumors is about 59 years [26]. The age distribution differs by tumor sites and histologic types, suggesting the likelihood of many distinct etiologic factors for the different histologic types. For instance, the peak of incidence in astrocytoma and glioblastoma is at the age of 65 to 74 years, and in oligodendroglioma is at the age of 35 to 44 years. Some of this variation may reflect different diagnostic practices and access to diagnosis in different age groups. It is likely that the duration of exposure required for malignant transformation, the number of genetic alterations required for disease development, or poorer immune surveillance with advanced age may contribute to susceptibility of different tumor types. Patients with GBM have the poorest survival in all age groups and within any histologic type. The pediatric (under age 20 years) and younger adult (age 20-44 years) populations have much better survival compared to older adults within each histologic type of primary malignant brain cancer. This study revealed that age 40-60 years old stroke patients have highest risk of developing brain tumors with an adjusted HR of 16.34 (95% CI: 4.45-62.13) (Table 4).

Gliomas affect about 40% more males than females [27]. A recent study from New York State showed that the sex differential (greater incidence in males) in GBM began to be evident around the age of menarche, was greatest around the age of menopause, and decreased thereafter, suggesting that female hormones may have a protective effect [28]. Any comprehensive theory regarding the distribution and causes of brain cancers should explain the biological and social factors that account for these consistent observations in sex differences. In this study, the female stroke patients have higher risk of developing brain tumors with an adjusted HR of 7.41 (95% CI: 3.30-16.64) (Table 4). It is believed that the incubation period of brain cancer is from months to years. However, in this analysis, the development of brain tumor is rapidly increased in the stroke group within one year compared to the control group (Figure 3). This short time period before malignant transformation suggests that brain tissue damage after stroke is a powerful risk for brain tumor development.

In review of two clinical GBM cases previously with stroke, one case is first side infarction, second right side infarction, but subsequent right side GBM occurrence. Another case is bilateral infarction but subsequent cerebellar vermis GBM formation. Why is the location of the subsequent GBM different from the previous stroke area? Faiz et al. [29] report that in response to focal ischemia, neural stem cells (NSCs) leave their niche in the subventricular zone (SVZ) and migrate to the damaged cortex, where they eventually differentiate into reactive astrocytes (RAs) that contribute to the glial scar surrounding the injury core. The RAs are a heterogeneous population of cells that play key roles in the CNS response to injury [30], such as restricting inflammation, preventing neuronal loss, and repairing the blood brain barrier [31]. So the RA may be in relation with the incidence of malignant glioma in the different location from the stroke site.

A number of previous studies have already described an increase in expression of the HIF-1α, with increasing malignancy grade from grades II to IV [32–34]. The possible mechanisms for developing brain cancer in stroke patients may arise from the overproduction of HIF-1α by ischemic brain tissue in stroke patients. HIF-1α is a nuclear transcription factor characterized as the master regulator of cellular oxygen homeostasis and is rapidly upregulated in response to hypoxia [35]. It activates the tissue survival pathways by inducing several key enzymes involved in cell metabolism (glucose transporter [GLUT]), angiogenesis (vascular endothelial growth factor [VEGF], vascular endothelial growth factor receptor 1 [VEGFR1], angiopoietin), and free radical scavenging (heme oxygenase 1 [HO-1]) [36]. The HIF-1α signaling is deeply involved in both pathological (hypoxia) and neural repairing (normoxia) pathways after stroke injury [37]. VEGF has been shown to modulate coupling of angiogenesis and neurogenesis; hence, it is an essential factor for regeneration [38, 39]. The high VEGF expression on tumor endothelium results in growth and proliferation of endothelial cells [40]. This correlates with tumor hypoxia and necrosis, as well as a poor overall clinical prognosis [41, 42]. Effective inhibition of VEGF in GBM decreases neo-vascularization, enhances blood vessel integrity, reduces cancer-associated edema, and results in both improved clinical performance and progression-free survival [42, 43]. In addition, both HIF-1α and VEGF can upregulate chemokine receptor CXCR4 in angiogenesis and cell invasion of GBM [44]. In this report, strong staining of HIF-1α was observed in 100% (2/2) of GBMs previously with stroke. In contrast, no staining of HIF-1α was seen in 100% (3/3) of GBMs previously without stroke and 100% (3/3) of grade I astrocytomas (Table 5). Compared with malignant glioma previously without stroke, HIF-1α expression significantly increased in GBM previously with stroke. These results indicate that HIF-1α may play an important role in stroke inducing the incidence of GBM.

**Table 5:** Patient characteristics and HIF-1α expression in stroke and non-stroke groups

Case	Age	M/F	Location	Stroke history	Pathology	HIF-1α expression
1	72	M	R temporal	10 years ago	GBM	strong
2	67	F	B cerebellar	5 years ago	GBM	strong
3	50	F	R temporal	no	GBM	no
4	53	F	R frontal	no	GBM	no
5	47	F	L parietal	no	GBM	no
6	18	M	L cerebellar	no	Grade I astrocytoma	no
7	27	M	R temporal	no	Grade I astrocytoma	no
8	23	M	L temporal	no	Grade I astrocytoma	no

M, male; F, female; R, right; L, left; B, bilateral.

However, several limitations in this study should be considered. The codes of stroke and cancer were taken from diagnoses described in the NHIRD. Thus, some coding errors should be considered. In addition, the issue of disease misclassifications could be due to the claim data about the ICD-9-CM diagnosis. Moreover, the disease severity scores and stages of tumor were not available in the database. These factors may be potential confounders for brain cancer. The personal backgrounds, including environmental factors, education level, marital status, diet, smoking, and alcohol consumption, were not listed in the NHIRD. However, these personal factors are not known risk factors of developing brain cancer; we could not estimate the effects from these personal backgrounds. Further studies should be conducted to evaluate these effects.

In conclusion, this study revealed increased risk of brain cancer among patients previously with stroke, indicating that stroke might be seen as an early predictor and contributor to brain cancers. Moreover, the expression of HIF-1α is associated with GBM patients previously with stroke. So the possible mechanism of the primary GBM development may be due to stroke via HIF-1α pathway. Physicians in clinical practice should be alerted to this association so that they can identify these patients earlier. However, further studies are needed to obtain more information and to explore the mechanisms underlying these relationships.

## MATERIALS AND METHODS

### Patients and specimens

The cohort was assembled from patients who were histologically diagnosed with GBM and who underwent surgery at the Department of Neurological Surgery, Chi-Mei Foundation Medical Center, between 2015 and 2016. Exclusion criteria included recurrence at presentation, pre-operative radiotherapy, chemotherapy or incomplete medical records. All medical records were reviewed retrospectively, according to the inclusion and exclusion criteria. The formalin-fixed, paraffin-embedded specimens from these patients were used for immunohistochemical (IHC) staining. The study was approved by the Ethics Committee of Chi Mei Foundation Medical Center. Written informed consent was obtained from the patients.

### Immunohistochemical (IHC) staining

Sections (4 μm) were deparaffinised in xylene and rehydrated. Antigen retrieval was performed using the heat-induced epitope retrieval method. Slides were boiled in antigen retrieval buffer (1 mM EDTA solution, adjusted to pH 8.0) in a pressure cooker until full pressure was reached, and maintained for another 90 sec. After the slides were cooled to room temperature, they were incubated with a mouse monoclonal antibody against human HIF-1α (Novus biological, NB100-123; 1:200) at 4°C overnight. Instead of the primary antibody, the negative control was incubated with phosphate-buffered saline (PBS; pH 7.4). The slides were washed twice with PBS for 5 min and tissues were followed by incubation with HRP-conjugated secondary antibodies (DAKO) for IHC staining. Immunoreaction products of IHC staining were visualized by the DAB chromogen system (DAKO). The slides were washed twice for 5 min with PBS and then counterstained with Mayer's haematoxylin, washed, dehydrated and cleared. Coverslips were sealed with neutral balsam. The percentage of positive cells was estimated using an image analysis system. The level of HIF-1α expression was determined independently by three pathologists. Each pathologist determined the percentage of positive cell nuclei in each field. Tissues were scored according to the percentage of positive immunostaining (P) as follows: no (<1%), weak (1-10%), and strong (>10%).

### Data sources

We conducted a population-based cohort study from 1996 to 2013 by analyzing the Longitudinal Health Insurance Database 2000 (LHID2000) of the National Health Insurance Research Database (NHIRD) of Taiwan, created by the National Health Insurance (NHI) program in 1996 (http://nhird.nhri.org.tw/en/). The NHI program is a nationwide healthcare system in Taiwan established in 1995, and its coverage rate exceeded 99% in 2007. The LHID2000 consists of one million randomly-sampled people corresponding to about 4% of all enrollees. There were no significant differences in demographic characteristics, including age, sex, or income between the selected sample and all enrollees (http://nhird.nhri.org.tw/en/).

The LHID2000 contains separate datasets for information on each member, including an encrypted personal identification number, gender, date of birth, the diagnostic codes using the International Classification of Diseases, Ninth Revision, Clinical Modification (ICD-9-CM), drug prescriptions, medical cost, medical care facilities, and specialty of caregivers, both in outpatient and hospitalization settings. All datasets are interlinked by the personal identification number of the patient (http://nhird.nhri.org.tw/en/).

### Study samples

Patients were excluded if they had any diagnosis codes of stroke (ICD-9-CM 430-436) or cancers ((ICD-9-CM code: 140-199) before January 1, 2000. For the stroke group, we selected in-hospital patients with a primary diagnosis of ICD-9-CM codes 430-436 between January 1, 2000 and December 31, 2013. All patients had to have undergone brain imaging such as brain CT (order code: 33067, 33068, 33069, 33070) or MRI (order code: 33084, 33085). The control group was a random sample with 1:4 matching for gender and age at time of enrollment to the stroke group.

On each member of the two study groups, we collected information including demographic characteristics, risk factors, and comorbidities, including diabetes mellitus (DM; ICD9-CM code: 250, 357.2, 362.0, 366.41), hypertension (HTN; ICD-9-CM code: 401–405), coronary artery disease (CAD; ICD-9-CM code: 410–414), chronic kidney disease (CKD; ICD-9-CM code: 582, 583, 585, 586, 588), and hyperlipidemia (ICD-9-CM code: 272).

All patients were followed until the earliest outcome: death, end of 2013, or diagnosis of various cancers by at least 3 outpatient service claims or 1 inpatient service claim with ICD-9-CM codes as follows: head and neck cancer (ICD-9-CM code: 140-149), digestive cancer (ICD-9-CM code: 150-159, 179, 185-189), respiratory cancer (ICD-9-CM code: 160-165), bone and skin cancer (ICD-9-CM code: 170-173,176), brain cancer (ICD-9-CM code: 191, 192.0-192.1). The ethical approval of this study was granted by the Institutional Review Board of the Ethics Committee of Chi Mei Medical Center (10410-E05). Access to the database used in this study was allowed by the Review Committee of the National Health Research Institutes (NHRI-104-112).

### Statistical analyses

All the data processing and statistical analysis were performed by using SAS statistical software (SAS Institute Inc., Version 9.3.1, Cary, NC, U.S.A.). Chi-square tests were used to evaluate the differences in categorical data, while Student's t tests were used for continuous variables. Kaplan-Meier analyses were used to calculate the cumulative incidence of cancer in the two groups, and log-rank tests were used to evaluate the differences between stroke patients and references patients. Cox regression models were constructed to evaluate the effects of risk factors for possible confounding factors, including DM, HTN, CAD, CKD, and hyperlipidemia. A two-tailed *p* value of < 0.05 was considered statistically significant.
